# Phylogenetic signals and predictability in plant–soil feedbacks

**DOI:** 10.1111/nph.16768

**Published:** 2020-07-31

**Authors:** Elizabeth M. Wandrag, Sarah E. Bates, Luke G. Barrett, Jane A. Catford, Peter H. Thrall, Wim H. van der Putten, Richard P. Duncan

**Affiliations:** ^1^ Institute for Applied Ecology University of Canberra Canberra ACT 2617 Australia; ^2^ School of Environmental and Rural Science University of New England Armidale NSW 2350 Australia; ^3^ Department of Terrestrial Ecology Netherlands Institute of Ecology (NIOO‐KNAW) PO Box 50 Wageningen 6700 AB the Netherlands; ^4^ CSIRO Agriculture and Food Canberra ACT 2601 Australia; ^5^ Department of Geography King’s College London London WC2B 4BG UK; ^6^ School of BioSciences University of Melbourne Melbourne Vic. 3010 Australia; ^7^ Laboratory of Nematology Wageningen University PO Box 8123 Wageningen 6700 ES the Netherlands

**Keywords:** biotic interactions, Brownian evolution, mutualisms, pairwise feedbacks, pathogens, plant–soil interactions, symbioses

## Abstract

- There is strong evidence for a phylogenetic signal in the degree to which species share co‐evolved biotic partners and in the outcomes of biotic interactions. This implies there should be a phylogenetic signal in the outcome of feedbacks between plants and the soil microbiota they cultivate. However, attempts to identify a phylogenetic signal in plant–soil feedbacks have produced mixed results.
- Here we clarify how phylogenetic signals could arise in plant–soil feedbacks and use a recent compilation of data from feedback experiments to identify: whether there is a phylogenetic signal in the outcome of plant–soil feedbacks; and whether any signal arises through directional or divergent changes in feedback outcomes with evolutionary time.
- We find strong evidence for a divergent phylogenetic signal in feedback outcomes. Distantly related plant species show more divergent responses to each other’s soil microbiota compared with closely related plant species. The pattern of divergence implies occasional co‐evolutionary shifts in how plants interact with soil microbiota, with strongly contrasting feedback responses among some plant lineages.
- Our results highlight that it is difficult to predict feedback outcomes from phylogeny alone, other than to say that more closely related species tend to have more similar responses.

## Introduction

Phylogenetic signal is the tendency for closely related species to share greater resemblance than species drawn randomly from a phylogenetic tree (Blomberg & Garland, 2002; Münkemüller *et al*., 2012). Phylogenetic signals arise when similarity between species is related to the time since their evolutionary divergence, or phylogenetic distance. Due to a longer shared evolutionary history, recently diverged species are more likely to share features in common than species that diverged in the more distant past (Harvey & Pagel, 1991). Phylogenetic signals can also arise in the relationships between species and the taxa with which they have strong co‐evolutionary interactions (Koyama *et al*., 2019). For example, as plant species diverge from each other in evolutionary time, their biotic partners (pests, pathogens and symbiotic mutualists) tend also to diverge such that closely related plant species share more co‐evolved biotic partners than distantly related plant species (Gilbert & Webb, 2007). A phylogenetic signal in the degree to which species share co‐evolved biotic partners should lead to a phylogenetic signal in the outcome of biotic interactions (Gilbert & Parker, 2016). This prediction is supported by empirical studies: closely related plant species tend to respond in similar ways when exposed to the same pathogens (Gilbert *et al*., 2015), fungal endophytes (Giauque *et al*., 2019) and symbiotic mutualistic soil microbes (Barrett *et al*., 2016; Hoeksema *et al*., 2018), relative to the responses of more distantly related species.

Much recent interest has focused on the co‐evolutionary relationships that plants form with soil microbiota (van der Putten, 2010; Crawford *et al*., 2019; Kandlikar *et al*., 2019), referred to as plant–soil feedbacks. Feedbacks arise because plant species cultivate specific soil microbiota, and soil microbiota in turn affect plant performance. These feedbacks can be positive (the soil microbiota cultivated by a plant species has a net positive effect on its growth relative to either sterilized soil or the soil microbiota cultivated by other plant species) or negative. Because feedbacks between plants and soil biota can differentially alter species performance and competitive ability (Bever, 2003), plant–soil feedbacks are thought to play an important role in community‐level processes such as plant species coexistence and invasion (Bonanomi *et al*., 2005; Bell *et al*., 2006; van der Putten *et al*., 2007). Consequently, interest has centred on predicting how plant species respond to both their own soil microbiota and the microbiota cultivated by other plant species. As with other biotic interactions, it is widely held that feedback outcomes should be predictable from plant species relatedness, implying a phylogenetic signal (Mehrabi & Tuck, 2015; Fitzpatrick *et al*., 2016).

**Fig. 1:**
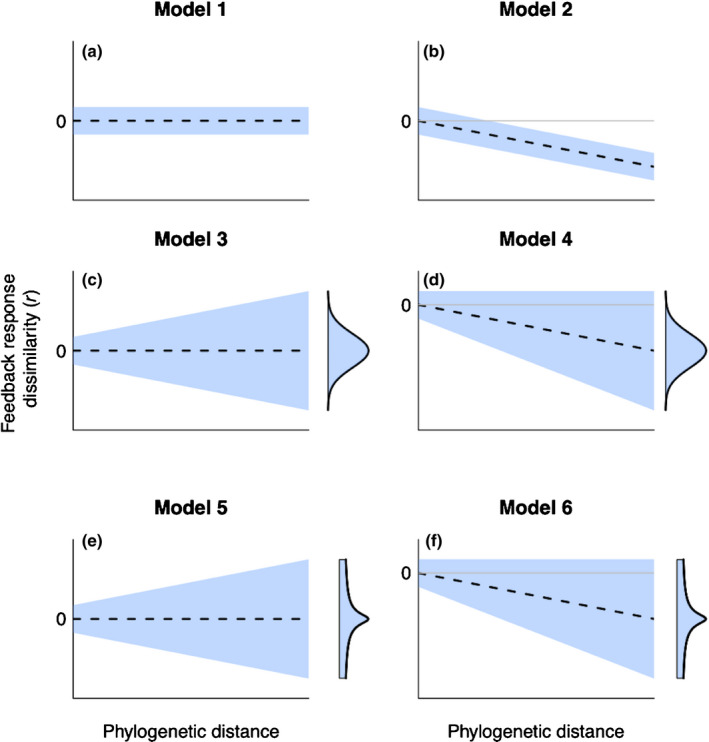
Phylogenetic signals arise when closely related species tend to be more similar (less dissimilar) than distantly related species. In (a–f) dashed lines show mean trend in dissimilarity with increasing phylogenetic distance (where dissimilarity can be in a positive or negative direction) and blue shading indicates variation in dissimilarity around the mean. In (a), (dis)similarity among species is the same, on average, regardless of phylogenetic distance, meaning there is no phylogenetic signal due to no change in mean dissimilarity and no change in variation with increasing phylogenetic distance. Panel (b) shows a phylogenetic signal arising from a directional trend in mean dissimilarity with increasing phylogenetic distance, resulting in more distantly related species being more dissimilar to each other (here, in a negative direction) relative to closely related species. In both (c) and (e), a phylogenetic signal arises due to an increase in variance but no directional trend, with more distantly related species being more likely to differ from each other in either direction relative to more closely related species. In (c), continuous gradual divergence over time leads to normally distributed responses while in (e), occasional major co‐evolutionary shifts lead to a heavy‐tailed distribution of responses. Panels (d) and (f) show a phylogenetic signal arising due to both an increase in variance and a directional trend, with a normally distributed (d) or heavy‐tailed (f) distribution of responses.

If there is a phylogenetic signal in plant–soil feedbacks, we expect closely related plant species to cultivate similar soil microbiota, and to respond in a similar way to each other’s microbiota, relative to more distantly related species. However, a phylogenetic signal in plant–soil feedbacks could arise in at least two ways, with different implications for how relatedness might predict feedback outcomes. First, a phylogenetic signal could result from a directional trend whereby plant species perform consistently better (positive feedback) or worse (negative feedback) in their own soil relative to other species’ soil with increasing phylogenetic distance (Fig. 1b). A directional trend implies that feedback strength and direction are predictable from plant species relatedness, which could have consequences for plant community structure. For example, because negative feedbacks can enhance plant species coexistence (Bonanomi *et al*., 2005), stronger negative feedbacks with increasing phylogenetic distance should favour plant communities with greater phylogenetic diversity (Crawford *et al*., 2019).

Evidence for a directional trend is mixed. Studies have variously found that species perform better, worse or much the same when exposed to soil microbiota cultivated by close compared to distant relatives (Dostal & Paleckova, 2011; Liu *et al*., 2012; Miller & Menalled, 2015; Mehrabi *et al*., 2015; Sweet & Burns, 2017; Kempel *et al*., 2018; Kuťáková *et al*., 2018; Wilschut *et al*., 2019), with meta‐analyses showing either no (Mehrabi & Tuck, 2015) or a slight negative directional trend (Crawford *et al*., 2019). However, soil feedback experiments usually involve whole soil communities, including both pathogens and symbiotic mutualists. While a higher specificity of pathogens compared with mutualists could result in a negative relationship between feedbacks and phylogenetic distance (see, for example, Crawford *et al*., 2019), there seems no compelling reason why the net effect of pathogens and mutualists on plant performance should generate a consistent directional trend in feedback responses with increasing phylogenetic distance (Jiang *et al*., 2020). Instead, the observed variation in directional trends could reflect aspects of the experimental design favouring pathogens or mutualists, or affecting plant species responses to those pathogens and mutualists.

Second, a phylogenetic signal can arise if feedback responses diverge over time but in no consistent direction (Fig. 1c,e). As with directional trends, there is evidence from empirical studies for divergent phylogenetic signals in feedback outcomes (Diez *et al*., 2010; Anacker *et al*., 2014; Fitzpatrick *et al*., 2016; Senior *et al*., 2018). A divergent phylogenetic signal is consistent with our understanding of plant–soil feedbacks, because it implies that both pathogens and mutualists can drive plant responses to soil biota. However, it changes our expectations about how predictable feedback outcomes are from knowledge of species relatedness. On the one hand, nondirectional divergence over time implies some degree of predictability, in that closely related species should respond in a similar way to each other’s microbiota. On the other hand, this pattern of divergence implies the strength and direction of feedbacks become more variable, and hence less predictable, among more distantly related species.

Nevertheless, if phylogenetic signals do arise though divergence over time, the pattern of divergence could provide insight into the underlying co‐evolutionary processes (Fig. 1c–f) and provide some predictability to feedback outcomes. For example, plant species could diverge gradually in their feedback responses over time due to the accumulation of many small changes in the way plants and soil microbiota interact. A process of cumulative gradual change is equivalent to a Brownian motion model of evolutionary change, which should generate approximately normally distributed feedback responses (Fig. 1c,d) with the variance in feedback response increasing in direct proportion to the phylogenetic distance between plant species (Harvey & Pagel, 1991; Elliot & Mooers, 2014). The nondirectional accumulation of gradual changes through evolutionary time implies that species will tend to drift apart, such that feedback responses become less predictable among more distantly related species. Alternatively, divergence could include occasional major shifts in the way plants and soil microbiota interact. These shifts could occur if certain plant lineages formed unique co‐adaptations with key pathogens or mutualists, such as nitrogen‐fixing bacteria or specialized mycorrhizae. Such lineage‐based shifts should result in a distribution of feedback responses different to that expected under continuous gradual change, potentially adding some predictability to feedback outcomes. For example, we might expect plant species in the same lineage to respond similarly to each other’s soil microbiota if they share a key co‐adapted mutualist, but to respond differently to the soil microbiota cultivated by plants in lineages lacking that mutualist. Occasional major co‐evolutionary shifts in some lineages should lead to feedback responses having a more peaked distribution with heavier tails relative to a model of continuous gradual change (Fig. 1e,f).

The varying ways in which a phylogenetic signal could arise might explain why it has proven difficult to identify a clear signal of relatedness in plant–soil feedbacks. Here, we aim to resolve this issue and gain insight into the nature of co‐evolutionary relationships between plants and soil microbiota. We take advantage of a major compilation of data from plant–soil feedback studies (Crawford *et al*., 2019) to assess the degree to which a phylogenetic signal arises through a directional and/or divergent trend in feedback responses and characterise the pattern of response divergence (e.g. Fig. 1).

## Materials and Methods

### The data

We used the data in Crawford *et al*. (2019), which is a compilation of plant–soil feedback data from multiple studies involving pairwise feedbacks where two plant species were grown in soil cultivated by both species. The data included estimates of the phylogenetic distance between each plant species pair, as well as details of experimental treatments, whether data were derived from glasshouse or field experiments, whether the plant species were from grassland or forest ecosystems and were native or not to those systems, and each species functional group, which included grasses, forbs and trees. The full dataset included studies that used whole soil communities and studies that used some fraction of the soil community, such as mycorrhizal fungi, in measuring feedback responses. We included only studies using whole soil communities because feedbacks involving a subset of the soil biota are likely to differ from those generated by whole soil communities, potentially obscuring the pattern we were interested in. Where whole soil communities were used, an inoculum of the whole soil was typically added to pots of sterile soil, with the proportion of whole soil inoculum relative to sterile soil ranging from less than 0.01 to 1. Adding a small amount of inoculum to sterilized soil is a technique used to introduce soil microbiota while minimising changes to species’ performance due to differences in abiotic soil properties. While abiotic feedbacks are expected to be small relative to biotic feedbacks (Crawford *et al*., 2019), feedback responses will reflect changes to both biotic and abiotic soil properties caused by the cultivating species, and phylogenetic signals could arise due to the differing response of species to both components.

The data we analysed comprised 968 feedbacks from 470 unique species pairs involving 165 species from 39 plant families. Consequently, over half of the 968 feedbacks involved replicates of a species pair, most associated with different experimental treatments that were reported separately in the dataset compiled by Crawford *et al*. (2019), such as feedbacks involving the same species pair measured in soil with different resource levels. To identify a phylogenetic signal in feedbacks, we considered each species pair in our analysis (see the section ‘Phylogenetic signal in plant–soil feedbacks’) to be an independent data point with multiple feedbacks per pair treated as pseudoreplicates (Hurlbert, 1984). We used the estimates of phylogenetic distance provided in the dataset.

### Estimating the similarity of species responses to soil microbiota

Crawford *et al*. (2019) raised the issue that differences in study design or methodology might confound comparisons among studies. For example, variation in factors such as soil type and nutrient status can alter plant responses to soil microbiota, potentially obscuring efforts to identify a phylogenetic signal. This problem can be overcome using data from pairwise plant–soil feedback experiments, allowing data from different experiments and studies to be compared directly (Crawford *et al*., 2019). We next describe a method to estimate the dissimilarity in feedback response when two plant species are exposed to two soil communities, regardless of the origin of those soil communities. We then show how a measure of feedback dissimilarity can be calculated using data from pairwise feedback experiments, and how this dissimilarity measure should change as a function of phylogenetic distance given different models of evolutionary divergence in feedback response.

Consider two soil microbial communities, 1 and 2. If we conduct a pairwise experiment where we grow two plant species, *A* and *B*, in association with each soil community, we can measure the relative performance of species in each soil as the log ratio of the biomass of species *A* to species *B* (or vice versa): $\log_{e}\left(\frac{A_{1}}{B_{1}}\right)$ and $\log_{e}\left(\frac{A_{2}}{B_{2}}\right)$, where *A*_1_ is the biomass of species *A* when grown with soil community 1. The log transformation ensures that a proportional difference in biomass has the same magnitude whether positive or negative.

If the two species respond in the same way to the different soil communities, we expect the two log ratios to be equal. That is, if the net effect of soil community 2 is to reduce (or increase) the biomass of species *A* by 20% relative to soil community 1, we expect the same proportional reduction (or increase) in biomass for species *B* if it responds the same way as species *A*. Because we measure relative differences, this holds regardless of any absolute difference in biomass between species *A* and *B*, and independent of the origin of the two soil communities. A difference in the log ratios indicates that plant species differ in their response to the two soil communities, with the magnitude of difference a measure of the dissimilarity in response: a large difference indicates a more divergent response. For example, relative to soil community 1, if the net effect of soil community 2 is to reduce the biomass of species *A* by 20% but reduce the biomass of species *B* by 10%, the difference in log ratio is 0.12. If the difference in response is more pronounced, such that the net effect of soil community 2 is to reduce the biomass of species *A* by 20% but increase the biomass of species *B* by 30% relative to soil community 1, the difference in log ratio increases to 0.49.

### Using data from pairwise plant–soil feedback experiments to calculate dissimilarity in response

Pairwise feedback experiments measure the performance of two species in association with their own soil microbiota and with the microbiota of the other species. We can calculate the dissimilarity in response, *r*, to soil microbiota cultivated by one species relative to that cultivated by the other as the difference in the log ratios of the biomass of species *A* and *B* in association with each soil microbiota:

(Eqn 1) $$r=\log_{e}\left(\frac{A_{a}}{B_{a}}\right)-\log_{e}\left(\frac{A_{b}}{B_{b}}\right)$$

where *A_a_* is the biomass of species *A* grown with its associated soil community *a*. The log transformation ensures that the magnitude of *r* is the same regardless of which species is chosen as the numerator and which as the denominator. However, for whichever species is chosen as the numerator, how we interpret the direction of *r* (positive or negative) depends on whether the numerator in the left‐hand log ratio denotes performance in conspecific (e.g. *A_a_*, as in Eqn 1) or heterospecific soil (e.g. *A_b_*). Specifying the numerator in the left‐hand log ratio as performance in conspecific soil means that a positive value of *r* is associated with species performing better overall in conspecific relative to heterospecific soil, which is the usual definition of a positive soil feedback. Equation 1 is the dissimilarity measure that Crawford *et al*. (2019) analysed for a directional trend in feedback outcomes with phylogenetic distance. Here, we use this measure to test for both a directional trend in feedback outcomes (values of *r* increasingly diverge from zero with greater phylogenetic distance in a consistent positive or negative direction) and a divergent trend in feedback outcomes (values of *r* increasingly diverge from zero but in no consistent direction), and quantify the pattern of divergence.

### Phylogenetic signal in plant–soil feedbacks

We used the dissimilarity response measure, *r*, to test for a phylogenetic signal in plant–soil feedbacks by comparing the fit of six models to the data, with the different models specifying different types of phylogenetic signal (Fig. 1). For each pairwise feedback, we used the estimates of *A_a_*, *A_b_*, *B_a_* and *B_b_* in Crawford *et al*. (2019) to calculate *r_ij_*, the dissimilarity response of the *i*th replicate for the *j*th species pair (with one to 11 replicates per species pair), using Eqn 1. We used the corresponding standard errors of the estimates to calculate the variance, $\sigma_{r_{\mathrm{ij}}}^{2}$, of each *r_ij_* using the formula in Crawford *et al*. (2019).

For each pairwise feedback, we assumed there was a true value for *r_ij_* but this had not been observed directly. Instead, each *r_ij_* was an estimate of the true value with uncertainty $\sigma_{r_{\mathrm{ij}}}^{2}$. To allow this uncertainty to propagate through the analysis, we modelled each *r_ij_* as sampled from a normal distribution with mean given by the true response value $r_{\mathrm{ij}}^{*}$:

$$r_{\mathrm{ij}}∼\text{Normal}\left(r_{\mathrm{ij}}^{*},\sigma_{r_{\mathrm{ij}}}^{2}\right)$$

To deal with nonindependence due to replicated species pairs, we modelled each $r_{\mathrm{ij}}^{*}$ as sampled from a distribution with a different mean dissimilarity response for each species pair, $r_{j}^{*}$, and variance estimated from the data, with the variance, $\sigma_{w}^{2}$, quantifying the variation in $r_{\mathrm{ij}}^{*}$ among replicates *within* species pairs:

$$r_{\mathrm{ij}}^{*}∼\text{Normal}\left(r_{j}^{*},\sigma_{w}^{2}\right)$$

We then used estimates of $r_{j}^{*}$ as the response variable in six models that specified different ways in which a phylogenetic signal could arise (Fig. 1). Our aim was to identify the model that best fitted the data as a basis for inferring the nature of the phylogenetic signal in plant–soil feedbacks.

### The models

Model 1 (equivalent to Fig. 1a) assumed no phylogenetic signal in the data by modelling the $r_{j}^{*}$ as drawn from a normal distribution with mean zero, implying no directional trend in dissimilarity with increasing phylogenetic distance, and constant variance, $\sigma^{2}$, which quantifies the variation in dissimilarity response *among* species pairs:

$$r_{j}^{*}∼\text{Normal}\left(0,\sigma^{2}\right)\;\left(\text{Model}\;1\right)$$

Model 2 (e.g. Fig. 1b) assumed a directional trend in mean response but constant variance:

$$r_{j}^{*}∼\text{Normal}\left(\beta t_{j},\sigma^{2}\right)\;\left(\text{Model}\;2\right)$$

where *t_j_* is the phylogenetic distance between the *j*th plant species pair, and β measures the tendency for the mean value of $r_{j}^{*}$ to become either increasingly positive or negative with increasing phylogenetic distance (Fig. 1b shows *r* becoming increasingly negative, but the model tests for shifts in either direction).

Model 3 (equivalent to Fig. 1c) assumed nondirectional divergence over time. Under a model in which feedback responses diverge gradually through incremental changes drawn from a random distribution, the sum of increments over time will follow a normal distribution with variance increasing in direct proportion to time since divergence: a Brownian motion model of evolutionary change used widely to model continuous trait variation (Harvey & Pagel, 1991; Elliot & Mooers, 2014). We specified this model as:

$$r_{j}^{*}∼\text{Normal}\left(0,\sigma^{2}+kt_{j}\right)\;\left(\text{Model}\;3\right)$$

which has mean zero, indicating no directional trend, and variance $\sigma^{2}+kt_{j}$, where *k* measures the rate of change in the variance of $r_{j}^{*}$ with increasing phylogenetic distance.

Model 4 (Fig. 1d) specified a phylogenetic signal resulting from both a directional trend (as in model 2) and gradual divergence in feedback responses over time (as in model 3):

$$r_{j}^{*}∼\text{Normal}\left(\beta t_{j},\sigma^{2}+kt_{j}\right)\;\left(\text{Model}\;4\right)$$

In contrast to models of continuous gradual divergence (models 3 and 4), gradual change coupled with occasional major co‐evolutionary shifts in some lineages could constrain feedback responses, leading to a more peaked distribution with more extreme outcomes, and thus heavier tails, than captured by a normal distribution (Elliot & Mooers, 2014). We modelled this outcome (model 5) using a three‐parameter Student’s *t* distribution, which is widely used to model heavy‐tailed continuous distributions (Anderson *et al*., 2017).

Model 5 (Fig. 1e) specified that response outcomes followed a Student’s *t* distribution with mean zero (no directional trend), scale parameter *s* ^2^ and parameter υ controlling the degree of kurtosis, with smaller values of υ implying a more heavy‐tailed distribution (more extreme values) with higher variance. We allowed the kurtosis, and hence the variance, to change with phylogenetic distance, specified by rate parameter *k*, with smaller values of *k* indicating a more heavy‐tailed distribution with higher variance:

$$r_{j}^{*}∼\text{Student’s}\;t\left(0,s^{2},\upsilon +kt_{j}\right)\;\left(\text{Model}\;5\right)$$

Model 6 (Fig. 1f) was the same as model 5 but allowed for a directional trend in mean response along with nongradual divergence through evolutionary time:

$$r_{j}^{*}∼\text{Student’s}\;t\left(\beta t_{j},s^{2},\upsilon +kt_{j}\right)\;\left(\text{Model}\;6\right)$$

### Are feedback responses more similar within families?

**Fig. 2:**
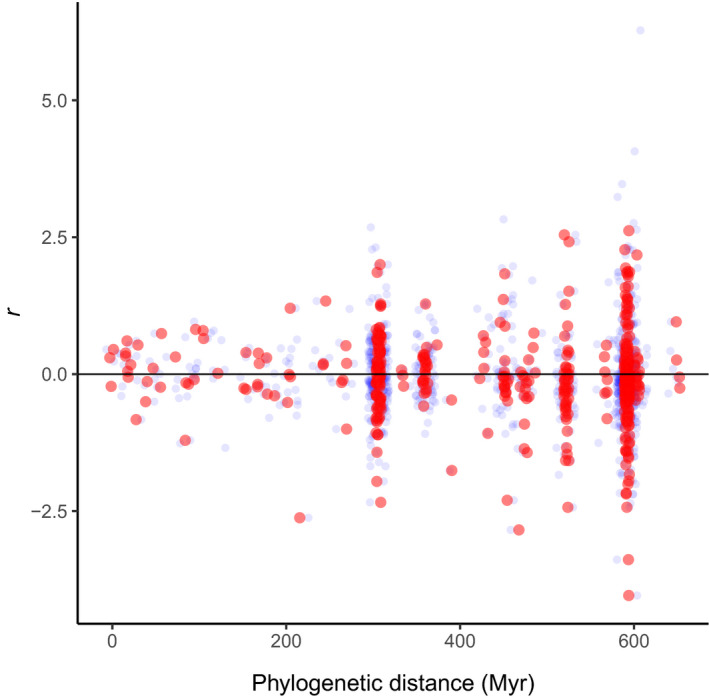
Values of *r*, a measure of feedback response dissimilarity (a value of zero means two species responded in the same way to their own and each other’s soil), plotted against phylogenetic distance. Blue circles are the data for 968 feedback responses from a compilation of pairwise plant–soil feedback experiments using whole soil communities (Crawford *et al*., 2019). Red circles are the mean feedback responses for each unique species pair (*n* = 470). The *x*‐axis values have been jittered so points are visible.

A heavy‐tailed distribution could arise if feedback responses in some plant lineages were constrained by unique co‐adaptations with soil microbiota. The variance of *r* appears to increase among species pairs separated by more than 300 million years (Myr; Fig. 2), which equates to an increase in variation among plant families relative to within plant families. Constraints at the family level might be expected, because we know that plant species in some families share unique co‐adaptations with soil biota (e.g. Fabaceae, Orchidaceae, Ericaceae), and recent evidence suggests that plant species are more responsive to mycorrhizal fungi cultivated by plants in the same family (Hoeksema *et al*., 2018). Such constraints could result in more similar feedback responses among plant species in the same family but greater differences in feedback response among species in different families. To examine this, we expanded model 6 to include terms estimating the mean feedback response between each pair of plant families for which responses had been measured, with the pairwise family means modelled hierarchically:

$$r_{j}^{*}∼\text{Student’s}\;t\left(\beta t_{j}+\alpha_{f},s^{2},\upsilon +kt_{j}\right)\;\left(\text{Model}\;7\right)$$

$$\alpha_{f}∼\text{Student’s}\;t\left(0,s_{\alpha }^{2},v_{\alpha }\right)$$

where $\alpha_{f}$ is a parameter estimating the deviation in response from the overall mean for the *f*th plant family pair, with the parameters modelled hierarchically as drawn from a Student’s *t* distribution with scale parameter $s_{\alpha }^{2}$ and kurtosis parameter $v_{\alpha }$, allowing the values to have a heavy‐tailed distribution. If unique co‐adaptations between plants and soil microbiota at the family level account for the heavy‐tailed distribution of feedback responses, then adjusting for pairwise family‐level differences should account for much of the phylogenetic signal in the data, leading to little or no increase in residual variance over time (i.e. after adjusting for pairwise family‐level differences, the pattern of residual variation should shift from Fig. 1f to Fig. 1a).

### Fitting the models

To allow the uncertainty at all levels to propagate through the analysis, we fitted the models in a Bayesian framework using jags (Just Another Gibbs Sampler; Plummer, 2003), specifying relatively uninformative priors to allow the data to drive parameter estimation. For continuous variables, we used normally distributed priors with mean zero and variance 10, and for variance terms we used uniformly distributed priors on the standard deviation in the range 0–10, which correspond to relatively uninformative priors. We ran the models with three chains for 10 000 iterations following a burn‐in of 1000 iterations and checked parameters for convergence using the Gelman–Rubin statistic (Gelman & Rubin, 1992), which was less than 1.1 for all parameters, indicating adequate convergence. We identified the best performing model using the approximate leave‐one‐out cross‐validation (LOO) criteria (Vehtari *et al*., 2017), which estimates the predictive accuracy of each model. LOO is considered an improvement on other information‐criterion‐based model selection measures such as the Akaike Information Criterion, Watanabe–Akaike Information Criterion and Deviance Information Criterion that are widely used to compare model performance (see Vehtari *et al*., 2017 for details). We compared models by calculating the difference in expected predictive accuracy (ΔLOO) between each model and the best‐fitting model on the deviance scale using the loo package in R (Vehtari *et al*., 2020), with smaller values implying a model with better predictive accuracy. The R code used to fit the models is provided in Supporting Information S1, along with code to draw all figures.

## Results

Fig. 2 plots the measure of response dissimilarity, *r*, against phylogenetic distance for the whole soil community feedback data in Crawford *et al*. (2019), revealing that more distantly related species (greater phylogenetic distance) appear to show greater variation in *r* values.

The six models shown in Fig. 1 (see the section ‘Are feedback responses more similar within families?’ for model 7) produced widely differing fits to the data as revealed by substantial differences in LOO values (Table 1). If we interpret differences in LOO values similarly to other information‐criterion measures, a difference in LOO > 10 indicates very strong support for one model relative to another (Lunn *et al*., 2012). Models 3–6 fit the data better than models 1 and 2, which specified constant variance among species pairs. This implies a phylogenetic signal arising, at least in part, through divergence in feedback responses over evolutionary time.

**Table 1:** Comparison of model performance with a lower LOO (leave‐one‐out cross‐validation) indicating a better performing model, and the difference in LOO (ΔLOO) between each model and the best fitting model.

Model	Model specification	Model summary	LOO	ΔLOO
7	$r_{j}^{*}∼\text{Student’s}\;t\left(\beta t_{j}+\alpha_{j},s^{2},\upsilon +kt_{j}\right)$	Family‐level shifts with directional trend	1550.3	0
6	$r_{j}^{*}∼\text{Student’s}\;t(\beta t_{j},s^{2},\upsilon +kt_{j})$	Co‐evolutionary shifts with directional trend	1592.6	42.3
5	$r_{j}^{*}∼\text{Student’s}\;t(0,s^{2},\upsilon +kt_{j})$	Co‐evolutionary shifts without directional trend	1593.2	42.8
4	$r_{j}^{*}∼\text{Normal}\left(\beta t_{j},\;\sigma^{2}+kt_{j}\right)$	Gradual divergence with directional trend	1615.4	65.0
3	$r_{j}^{*}∼\text{Normal}\left(0,\sigma^{2}+kt_{j}\right)$	Gradual divergence without directional trend	1616.6	66.3
2	$r_{j}^{*}∼\text{Normal}\left(\beta t_{j},\sigma^{2}\right)$	Constant variance with directional trend	1635.4	85.1
1	$r_{j}^{*}∼\text{Normal}\left(0,\;\sigma^{2}\right)$	Constant variance without directional trend	1637.2	86.8

$r_{j}^{*}$ is the mean dissimilarity response for the *j*th species pair. β measures the tendency for the mean value of $r_{j}^{*}$ to become increasingly positive or negative with increasing phylogenetic distance (*t_j_*). For normal distributions, $\sigma^{2}$ is the variance in response among species pairs, which is either constant or changing with phylogenetic distance at a rate estimated by parameter *k*. For Student’s *t* distributions, $s^{2}$ is a scale parameter and υ is a parameter controlling the degree of kurtosis, which changes with phylogenetic distance at a rate estimated by parameter *k*.

**Fig. 3:**
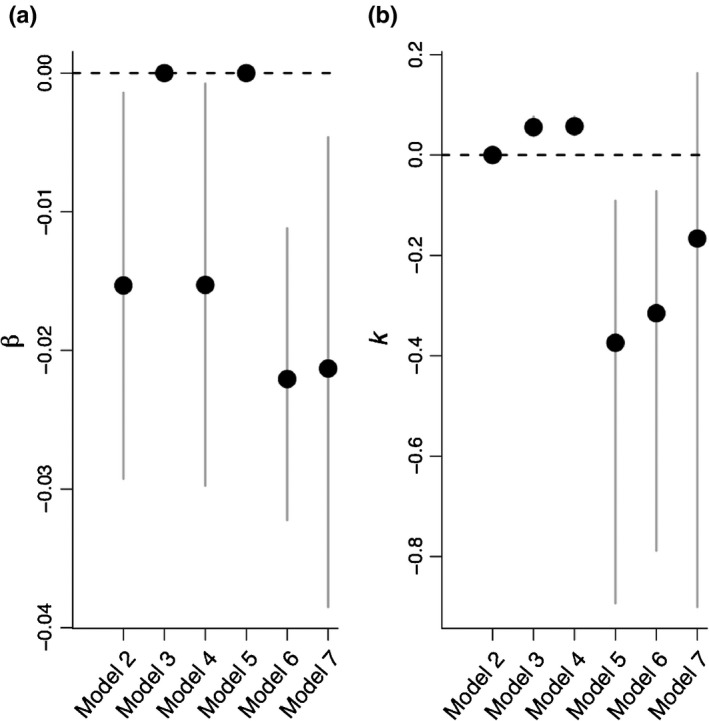
β (a) and *k* (b) parameter estimates for models 2–7 (model 1 did not include β or *k*) where β measures the tendency for feedback outcomes to become increasingly positive or negative with increasing phylogenetic distance and *k* measures the rate of change in the variance of feedback outcomes. Here, the estimates for β and *k* assume that one unit of phylogenetic distance equates to 100 Myr. The value of β was set to zero in models 3 and 5 (no directional trend), and the value of *k* was set to zero in model 2 (constant variance). For normal distributions (models 3 and 4), positive *k* values imply increasing variance in feedback responses with increasing phylogenetic distance. For Student’s *t* distributions (models 5–7), negative *k* values imply increasing kurtosis and increasing variance in feedback responses with increasing phylogenetic distance. Bars represent 95% credible intervals.

**Fig. 4:**
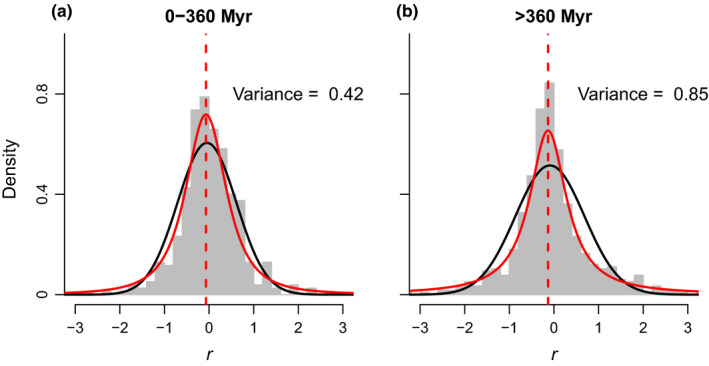
Density histograms of feedback response *r* (grey shaded bars) for phylogenetic distances involving feedbacks among species in the same family (a) and feedbacks among species in different families (b). The fit of model 4 (assuming a normal distribution of responses) to the density data at the median phylogenetic distance for data in the range is shown as a black line; the fit of model 6 (assuming a Student’s *t* distribution of responses) is shown as a red line, with the red dashed line showing the mean for model 6. Numbers to the right of the histograms are the variances of the data in each group.

Among the models specifying that feedback responses diverged over time (models 3–6), models 5 and 6 were the best performing, with both having similar predictive accuracy. Models 5 and 6 specified heavy‐tailed distributions and, for model 6, the parameter estimates identified a clear negative directional trend (a negative β parameter; Fig. 3) and increasing kurtosis with greater phylogenetic distance (a negative *k* parameter; Fig. 3). Comparing the fit of model 4 (the best‐fitting of the models that specified a normal distribution) with model 6 revealed that the better fit of model 6 to the data was due to the distribution of feedback responses being more peaked and having heavier tails than could be accommodated by the normal distribution specified in model 4, especially at large phylogenetic distances (Fig. 4).

While the negative β parameter for model 6 (Fig. 3; see also Crawford *et al*., 2019) implied that feedback responses tended to be more negative among plant species separated by a greater phylogenetic distance, this negative trend was of small magnitude relative to the increase in variance with increasing phylogenetic distance due to nondirectional divergence. The β parameter for model 6 implies that a 600‐Myr increase in phylogenetic distance between plant species results in the mean value of *r* declining by about 0.13, which is of much smaller magnitude than the shifts that result from an increase in variance over an equivalent time span (Fig. 2).

### Are feedback responses more similar within families?

**Fig. 5:**
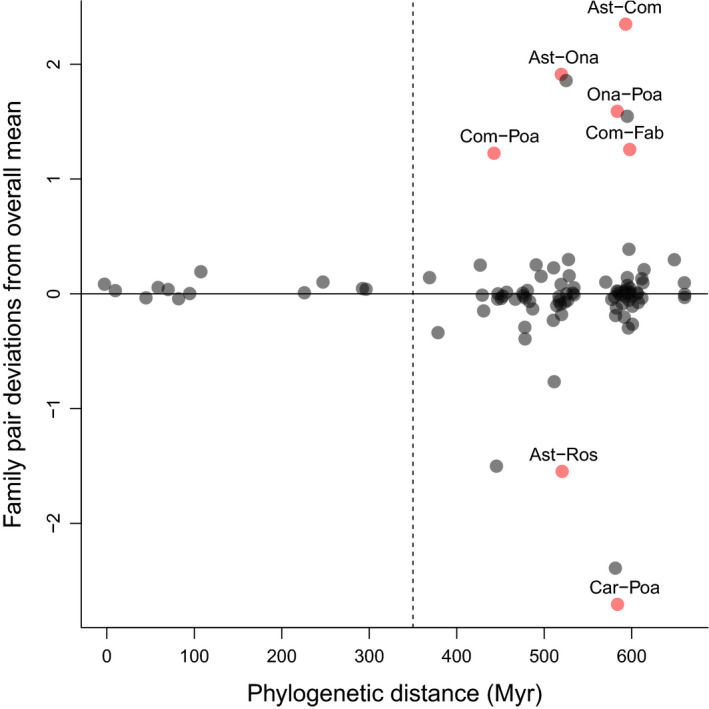
Estimates of the mean value of feedback response *r* for each family pair (model 7), expressed as a deviation from the overall mean, as a function of the mean phylogenetic distance between the species pairs in each family pair. Estimates of mean feedback response between species in the same family are to the left of the dashed line, and estimates of mean feedback response between species in different families are to the right. Red circles identify family pairs where the mean response differed from zero as judged by 95% credible intervals. Labels above the red circles are abbreviated names for the family pairs: Ast‐Com, Asteraceae–Commelinaceae (*n* = 15); Ast‐Ona, Asteraceae–Onagraceae (*n*  =  2); Ast‐Ros, Asteraceae–Rosaceae (*n*  =  2); Car‐Poa, Caryophyllaceae–Poaceae (*n*  =  2); Com‐Fab, Commelinaceae–Fabaceae (*n*  =  2); Com‐Poa, Commelinaceae–Poaceae (*n*  =  19); Ona‐Poa, Onagraceae–Poaceae (*n* = 8); *n*, the number of feedback responses in each family pair.

The distribution of feedback responses was consistent with divergence that involved occasional major shifts rather than continuous gradual change through evolutionary time (Elliot & Mooers, 2014), an outcome that could arise if feedback responses in some plant lineages became constrained by unique co‐adaptations with soil microbiota. Model 7 attempted to accommodate this, and had much better predictive accuracy than other models (Table 1). The magnitude of parameter *k* (measuring the change in kurtosis with phylogenetic distance) in model 7 was substantially less than in model 6, with 95% credible intervals that overlapped zero (Fig. 3). This implies that much of the increase in variance with increasing phylogenetic distance in model 6 could be accounted for by the pairwise family‐level estimates in model 7. The family‐level mean estimates for *r* in model 7 identified seven family pairs that differed significantly from the overall mean in their feedback responses (Fig. 5).

## Discussion

Plant–soil feedbacks can influence plant species performance and competitive ability, with implications for community assembly (Bever, 2003; Bonanomi *et al*., 2005). Consequently, considerable effort has been invested in understanding how plant species respond to their own soil biota and to soil biota cultivated by other species, including whether feedback responses can be predicted from plant species relatedness. Despite evidence that both the identity of biotic partners and the response of plant species to those partners are linked to phylogenetic relatedness (Barrett *et al*., 2016; Hoeksema *et al*., 2018; Giauque *et al*., 2019), attempts to identify a phylogenetic signal in feedback responses have produced mixed results. Using an extensive dataset compiled from plant–soil feedback studies (Crawford *et al*., 2019), we show that: there is a strong phylogenetic signal in plant–soil feedbacks; the phylogenetic signal arises primarily through nondirectional divergence of feedback responses over time with a slight tendency for responses to become more negative with greater phylogenetic distance (see also Crawford *et al*., 2019); and the pattern of divergence is consistent with occasional major co‐evolutionary shifts between plants and soil microbes rather than continuous gradual divergence.

Much research has examined whether there is a directional trend in feedback responses linked to phylogenetic relatedness. This is due largely to the putative importance of negative feedbacks in promoting coexistence and invasion, and positive feedbacks in promoting dominance by single species (Mehrabi & Tuck, 2015; Fitzpatrick *et al*., 2016; Kempel *et al*., 2018; Kuťáková *et al*., 2018). Our findings reiterate those of Crawford *et al*. (2019) in showing some evidence for a slight negative trend in feedback response with increasing phylogenetic distance. Such an outcome should favour coexistence among more distantly related species and thus promote communities with greater phylogenetic diversity (Bonanomi *et al*., 2005).

Nevertheless, our analysis highlights that any negative trend in feedback outcomes is slight compared with the overall increase in variance due to divergence in both directions over time. An increase in the variance of feedback responses over evolutionary time based on data from multiple studies is consistent with our understanding of plant–soil feedbacks, where the net effect of pathogens, mutualists and other components of the soil biota does not consistently alter plant performance in a particular direction (Jiang *et al*., 2020). Strong directional trends should only arise in specific situations where there are compelling reasons to expect a disproportionate influence of either pathogens or mutualists on focal species (e.g. Liu *et al*., 2012). The slight negative trend we observe could reflect a higher specificity of soil pathogens relative to soil mutualists, which could result in plants benefiting more through the loss of pathogens in soils of more distantly related species, relative to the cost of losing mutualists. The difference between what theory might predict about phylogenetic signals in specific situations or case studies and what theory predicts when integrating across data from multiple studies may be one reason why has proven difficult to identify a clear phylogenetic signal in the outcome of plant–soil feedbacks.

The increase in the variance of feedback responses due to divergence in both directions over evolutionary time implies that close relatives tend to respond to each other’s soil microbiota in similar ways, but that the magnitude and direction of feedback responses become more variable with greater phylogenetic distance. Consequently, it may only be possible to predict feedback outcomes with any accuracy among closely related species: phylogenetic distance is of less help in predicting the response among distantly related species.

Much of the increase in variability in feedback responses over evolutionary time was due to more extreme values than expected under a model of gradual divergence. This is consistent with major shifts associated with some plant lineages being constrained by co‐evolution with specialist microbiota. Such lineages should disproportionately benefit from escaping specialist natural enemies or disproportionately suffer from losing specialist mutualists, an outcome known to occur in some plant families. For example, the Orchidaceae (orchids) and Ericaceae (heaths) form specialized associations with orchid and ericoid mycorrhizal fungi, Fabaceae (legumes) rely on soil bacteria (rhizobia) for nitrogen fixation, and Poaceae (grasses) cultivate distinct microbial communities and are more responsive to those communities than other life‐forms (Hoeksema *et al*., 2010; Davison *et al*., 2020). In the data we analysed, seven family pairs had more extreme feedback responses than average, which included the families Fabaceae and Poaceae (Fig. 5; there were no Orchidaceae in the data). While it is important not to over‐interpret these results, because most between‐family comparisons involved relatively few species and feedback responses, modelling the variation associated with family‐level mean responses (Fig. 5) explained much of the increase in variation in feedback responses with increasing phylogenetic distance, leaving a weaker residual phylogenetic signal (parameter *k* was much closer to zero in model 7; Fig. 3). Hence, increasing divergence in feedback response with greater phylogenetic distance could be largely explained by the differing response of species in certain families to the microbiota associated with species in other families. Understanding variation in feedback responses within and among families may be one way to increase the predictability of feedback outcomes among more distantly related species.

### Conclusions

While relatedness can help to predict the outcome of some biotic interactions (Parker *et al*., 2015; Bufford *et al*., 2016), attempts to predict how plant species will respond to each other’s soil microbiota based on relatedness have produced mixed results. We have clarified how phylogenetic signals in plant–soil feedback outcomes could arise and used a recent compilation of data to quantify the nature of the phylogenetic signal. Our results reiterate other studies that provide evidence for, at best, a weak directional trend and highlight that knowledge of plant species relatedness is most likely a weak predictor of community‐level outcomes for plant–soil feedbacks. Our results indicate that it is difficult to predict how species will respond to each other’s soil microbiota from a knowledge of the phylogenetic distance between plant species alone, other than to say that more closely related species tend to have more similar responses. Nevertheless, this apparent loss in predictability could be offset by a divergence pattern that suggests feedbacks become constrained in some lineages by co‐evolution with specialist mutualists or enemies. If so, feedback outcomes among distantly related species might be predictable from knowledge of the lineages involved and how species in those lineages respond to each other’s soil biota (e.g. Fig. 5). Identifying families for which feedback responses have been constrained by co‐evolution with specialist soil microbiota and examining feedback outcomes for species within and among those families could improve our ability to predict outcomes.

## Author contributions

EMW, RPD and SEB conceived the idea, following discussion with LGB, JAC, PHT and WHvdP. EMW and RPD designed the methodology. RPD designed and performed the analyses. EMW and RPD led the writing of the manuscript with critical input from SEB, LGB, JAC, PHT and WHvdP. All authors gave final approval for publication.

## Supporting information

Please note: Wiley Blackwell are not responsible for the content or functionality of any Supporting Information supplied by the authors. Any queries (other than missing material) should be directed to the *New Phytologist* Central Office.

**Notes S1** R code used to perform the analyses and draw the figures.

## Data Availability

Data are from Crawford *et al*. (2019). *Figshare*. https://doi.org/10.6084/m9.figshare.7985195.v1. The code used to perform the analyses and draw Figs 1–5 is given Supporting Information Notes S1.
