# Supplementary material for: Phylogenetic signals and predictability in plant–soil feedbacks
Source: New Phytol. 2020 Jul 31;228(4):1440–9. doi: 10.1111/nph.16768 (PMC7689780; doi:10.1111/nph.16768)
Supplement: Supplementary file 1 — Notes S1 R code used to perform the analyses and draw the figures. [file NPH-228-1440-s001.pdf]

New Phytologist Supporting Information
Article title: Phylogenetic signals and predictability in plant-soil feedbacks
Authors: Elizabeth M. Wandrag, Sarah E. Bates, Luke G. Barrett, Jane A. Catford, Peter H. Thrall, Wim H. van der Putten and Richard P. Duncan
Article acceptance date: 12 June 2020

Supporting Information Notes S1 - R code used to perform the analyses and draw the figures.

R Code to draw Figure 1 on pages 1 – 3.
R code used to perform the analyses and draw Figures 2 – 5 on pages 4 – 16.

FIGURE 1

```
library(tidyverse)

setwd("")
xx <- 1:1000
cm <- 1.1

#########################################################################################
library(tidyverse)

xx <- 1:1000

cm <- 1.1

#########################################################################################

pdf("Figure 1.pdf")
par(mar = c(2, 4, 3, 1), fig = c(0, 0.45, 0.65, 1))
yl <- c(-80, 80)
yy <- rep(0, length(xx))

plot(yy ~ xx, ylim = yl, xaxt = "n", yaxt = "n", bty = "l", type = "n",
    xlab = "", ylab = "", xaxs = "i",
    main = "Model 1", cex.main = cm)
  polygon(x = c(0, 1000, 1000, 0), y = c(-15, -15, 15, 15), border = NA, col=rgb(102/255, 178/255,
255/255, 0.5))
  abline(h = 0, lwd = 2, lty = 2)
  text(-50, 0, "0", cex = 1, xpd = NA)
  title(ylab = "", mgp = c(1.5, 1, 0), cex.lab = 1)
  mtext(expression(bold(A)), side=3, adj=0, line=1, cex = 1.2)

#########################################################################################
par(fig = c(0.45, 0.9, 0.65, 1), new = TRUE)
yy <- -0.05 * xx
plot(yy ~ xx, ylim = yl, xaxt = "n", yaxt = "n", bty = "l", type = "n",
    xlab = "", ylab = "", xaxs = "i",
    main = "Model 2", cex.main = cm)
  polygon(x = c(0, 1000, 1000, 0), y = c(-15, -65, -35, 15), border = NA, col=rgb(102/255, 178/255,
255/255, 0.5))
  lines(yy ~ xx, lwd = 2, lty = 2)
```

```
  abline(h = 0, col = "grey")
   text(-50, 0, "0", cex = 1, xpd = NA)
   title(xlab = "", mgp = c(1.5, 1, 0), cex.lab = 1.4)
   mtext(expression(bold(B)), side=3, adj=0, line=1, cex = 1.2)

##################################################################################
 par(fig = c(0, 0.45, 0.35, 0.7), new = TRUE)
 plot(yy ~ xx, ylim = yl, xaxt = "n", yaxt = "n", bty = "l", type = "n",
     xlab = "", ylab = "", xaxs = "i",
     main = "Model 3", cex.main = cm)
  polygon(x = c(0, 1000, 1000, 0), y = c(-15, -65, 65, 15), border = NA, col=rgb(102/255, 178/255,
255/255, 0.5))
  abline(h = 0, lwd = 2, lty = 2)
   text(-50, 0, "0", cex = 1, xpd = NA)
   title(ylab = "Feedback response \n dissimilarity (r)", xlab = "", mgp = c(1.5, 1, 0), cex.lab = 1)
   mtext(expression(bold(C)), side=3, adj=0, line=1, cex = 1.2)

 par(fig = c(0.33, 0.53, 0.37, 0.68), new = TRUE)
  y2 <- seq(-3.5, 3.5, 0.01)
  x2 <- dnorm(y2)
  plot(y2 ~ x2, type = "l", xaxt = "n", yaxt = "n", xlab = "", ylab = "", bty = "n", lwd = 2, xlim = c(0, 0.4))
  polygon(c(0, x2), c(min(y2), y2), col=rgb(102/255, 178/255, 255/255, 0.5))

##################################################################################
 par(fig = c(0.45, 0.9, 0.35, 0.7), new = TRUE)
 yy <- 50 + -0.05 * xx
 plot(yy ~ xx, ylim = yl, xaxt = "n", yaxt = "n", bty = "l", type = "n",
     xlab = "", ylab = "", xaxs = "i",
     main = "Model 4", cex.main = cm)
  polygon(x = c(0, 1000, 1000, 0), y = c(35, -65, 65, 65), border = NA, col=rgb(102/255, 178/255,
255/255, 0.5))
  lines(yy ~ xx, lwd = 2, lty = 2)
  abline(h = 50, col = "grey")
   text(-50, 50, "0", cex = 1, xpd = NA)
   title(ylab = "", xlab = "", mgp = c(1.5, 1, 0), cex.lab = 1)
   title(ylab = "", xlab = "", mgp = c(1, 1, 0), cex.lab = 1)
   mtext(expression(bold(D)), side=3, adj=0, line=1, cex = 1.2)

 par(fig = c(0.77, 0.97, 0.37, 0.68), new = TRUE)
  y2 <- seq(-3.5, 3.5, 0.01)
  x2 <- dnorm(y2)
  plot(y2 ~ x2, type = "l", xaxt = "n", yaxt = "n", xlab = "", ylab = "", bty = "n", lwd = 2, xlim = c(0, 0.4))
  polygon(c(0, x2), c(min(y2), y2), col=rgb(102/255, 178/255, 255/255, 0.5))

##################################################################################
 par(fig = c(0, 0.45, 0, 0.35), new = TRUE)
 plot(yy ~ xx, ylim = yl, xaxt = "n", yaxt = "n", bty = "l", type = "n",
     xlab = "", ylab = "", xaxs = "i",
     main = "Model 5", cex.main = cm)
  polygon(x = c(0, 1000, 1000, 0), y = c(-15, -65, 65, 15), border = NA, col=rgb(102/255, 178/255,
255/255, 0.5))
```

```
  abline(h = 0, lwd = 2, lty = 2)
   text(-50, 0, "0", cex = 1, xpd = NA)
   title(ylab = "", xlab = "Phylogenetic distance", mgp = c(1, 1, 0), cex.lab = 1)
   mtext(expression(bold(E)), side=3, adj=0, line=1, cex = 1.2)

 par(fig = c(0.33, 0.53, 0.02, 0.33), new = TRUE)
  y2 <- seq(-3.5, 3.5, 0.01)
  x2 <- dt(y2, df = 0.1)
  plot(y2 ~ x2, type = "l", xaxt = "n", yaxt = "n", xlab = "", ylab = "", bty = "n", lwd = 2, xlim = c(-0.1,
0.15))
  polygon(c(min(x2)-0.04, x2, min(x2)-0.04), c(-3.5, y2, 3.5), col=rgb(102/255, 178/255, 255/255,
0.5))
  lines(y2 ~ x2, lwd = 2)

###############################################################################
 par(fig = c(0.45, 0.9, 0, 0.35), new = TRUE)
 yy <- 50 + -0.05 * xx
 plot(yy ~ xx, ylim = yl, xaxt = "n", yaxt = "n", bty = "l", type = "n",
     xlab = "", ylab = "", xaxs = "i",
     main = "Model 6", cex.main = cm)
  polygon(x = c(0, 1000, 1000, 0), y = c(35, -65, 65, 65), border = NA, col=rgb(102/255, 178/255,
255/255, 0.5))
  lines(yy ~ xx, lwd = 2, lty = 2)
  abline(h = 50, col = "grey")
   text(-50, 50, "0", cex = 1, xpd = NA)
   title(ylab = "", xlab = "Phylogenetic distance", mgp = c(1, 1, 0), cex.lab = 1)
   mtext(expression(bold(F)), side=3, adj=0, line=1, cex = 1.2)

 par(fig = c(0.77, 0.97, 0.02, 0.33), new = TRUE)
  y2 <- seq(-3.5, 3.5, 0.01)
  x2 <- dt(y2, df = 0.1)
  plot(y2 ~ x2, type = "l", xaxt = "n", yaxt = "n", xlab = "", ylab = "", bty = "n", lwd = 2, xlim = c(-0.1,
0.15))
  polygon(c(min(x2)-0.04, x2, min(x2)-0.04), c(-3.5, y2, 3.5), col=rgb(102/255, 178/255, 255/255,
0.5))
  lines(y2 ~ x2, lwd = 2)

dev.off()
```

ANALYSES AND FIGURES 2 – 5.

```
 library(tidyverse)
 library(jagsUI)
 library(loo)

 set.seed(456)
 setwd("")

# read in data
 dat <- read.csv("Crawford et al Supplementary Table 1.csv")
 glimpse(dat)

# include only whole soils
 table(dat$Inoculant.type)
 dat <- filter(dat, Inoculant.type == "WholeSoil")

# dissimilarity measure (r) calculations
 dat <- dat %>%
     mutate(ya = AinA.mean / BinA.mean,
         yb = AinB.mean / BinB.mean,
         rsum = log(ya) - log(yb),
         v1 = (AinA.se * sqrt(AinA.N))^2 / (AinA.N * AinA.mean^2),
         v2 = (BinA.se * sqrt(BinA.N))^2 / (BinA.N * BinA.mean^2),
         v3 = (AinB.se * sqrt(AinB.N))^2 / (AinB.N * AinB.mean^2),
         v4 = (BinB.se * sqrt(BinB.N))^2 / (BinB.N * BinB.mean^2),
         var.rsum = v1 + v2 + v3 + v4)

#########################################################################################
# standardise order for species and family pairs
 dat <- dat %>%
     mutate(fa = as.character(Family.A),
         fb = as.character(Family.B),
         test = ifelse(fa < fb, 1, 0),
         ff = ifelse(test == 1, paste(fa, fb), paste(fb, fa)),
         sa = as.character(Species.A),
         sb = as.character(Species.B),
         test = ifelse(sa < sb, 1, 0),
         ss = ifelse(test == 1, paste(sa, sb), paste(sb, sa)),
         same_fam = ifelse(fa == fb, 1, 0))

# range of within and among-family phylogenetic distances
 tapply(dat$Phylogenetic.distance, dat$same_fam, range)
 summary(dat$Phylogenetic.distanc[dat$same_fam == 1])
 summary(dat$Phylogenetic.distanc[dat$same_fam == 0])

#########################################################################################
 glimpse(dat)

# table of species pairs
 table(table(dat$ss))
```

```
# number of species pairs
  sum(table(table(dat$ss)))

# list of species names
  s1 <- dat$Species.A
  s2 <- dat$Species.B
  spp_list <- c(s1, s2)
  spp_list <- spp_list[duplicated(spp_list) == FALSE]
  length(spp_list)

# list of family names
  f1 <- dat$fa
  f2 <- dat$fb
  fam_list <- c(f1, f2)
  fam_list <- fam_list[duplicated(fam_list) == FALSE]
  length(fam_list)


# rsum is equivalent to rrIs
  par(mfrow = c(2, 2))
  plot(rsum ~ rrIs, data = dat)
  plot(dat$var.rsum ~ dat$Variance.rrIs.)

# calculate mean rsum for each species pair
  m.dat <- dat %>%
        group_by(ss, Phylogenetic.distance) %>%
        summarise(rsum = mean(rsum))

  dim(m.dat)

# plot of averaged species pair values
  ggplot(m.dat, aes(y = rsum, x = jitter(Phylogenetic.distance, 200))) +
    geom_point(size = 3, alpha = 0.2) +
    geom_hline(yintercept = 0) +
    ylab(expression(italic(r))) +
    xlab("\nPhylogenetic distance (myr)") +
    geom_vline(xintercept = 360) +
    theme_classic(16)

# plot of all data
  ggplot(dat, aes(y = rsum, x = jitter(Phylogenetic.distance, 200))) +
    geom_point(size = 3, alpha = 0.2) +
    geom_hline(yintercept = 0) +
    ylab(expression(italic(r))) +
    xlab("\nPhylogenetic distance (myr)") +
    geom_vline(xintercept = 360) +
    theme_classic(16)

# Figure 2
# plot of all data and averaged values
```

```
  pdf("Figure 2.pdf")

  ggplot(dat, aes(y = rsum, x = jitter(Phylogenetic.distance, 200))) +
    geom_point(size = 2, alpha = 0.1, colour = "blue") +
    geom_point(data = m.dat, aes(y = rsum, x = jitter(Phylogenetic.distance, 50)), size = 3, colour =
"red", alpha = 0.5) +
    geom_hline(yintercept = 0) +
    ylab(expression(italic(r))) +
    xlab("\nPhylogenetic distance (myr)") +
    geom_vline(xintercept = 360) +
    theme_classic(16)

  dev.off()

##########################################################################################
# data for analysis

# indicator variable for each species pair
  ss <- as.numeric(factor(dat$ss))
  N_ss <- max(ss)

# phylogenetic distance per 100 myr for each species pair
  tt <- tapply(dat$Phylogenetic.distance, factor(dat$ss), mean) / 100

  rsum <- dat$rsum
# standard error of rsum as precision
  prec.rsum <- ifelse(dat$var.rsum == 0, 100, 1 / dat$var.rsum)
  N <- length(rsum)

  niter = 11000
  nburn = 1000

# in calculating LOO values we have to decide on which part of the model to define as the likelihood.
# This determines which data we want to treat as the samples for defining predictive accuracy.
# Here we use the individual rij for each of the 968 feedbacks (rsum in the code below) as the data,
with its associated likelihood

##########################################################################################
# Model 1

  mod <- "model {
    for(i in 1:N) {
      rsum[i] ~ dnorm(rtrue[i], prec.rsum[i])                    # data are drawn from a distribution with
true mean = rtrue
      rtrue[i] ~ dnorm(mu[i], tau[1])
      mu[i] <- b.ss[ss[i]]                                # average value for each species pair
      loglik[i] <- logdensity.norm(rsum[i], rtrue[i], prec.rsum[i])  # log likelihood for each rsum for LOO
calculation
    }
```

```
  for(i in 1:N_ss) {                              # model the averaged species pair data
    b.ss[i] ~ dnorm(mu.ss[i], tau[2])
    mu.ss[i] <- 0
  }

  for(i in 1:2) {                                 # variance terms
    tau[i] <- 1 / sigma2[i]
    sigma2[i] ~ dunif(0, 10)
  }

 }"


 write(mod, "model.txt")
 mod1 <- jags(model = "model.txt",
         data = list(rsum = rsum, tt = tt, N = N, prec.rsum = prec.rsum,
                 ss = ss, N_ss = N_ss),
         param = c("sigma2", "loglik"),
         n.chains = 3,
         n.iter = niter,
         n.burnin = nburn,
         parallel = T)

 mod1.sum <- mod1$summary
 mod1.sum[1:10, c(1,2,3,5,7,8,9)]

####################################################################################
# Model 2

 mod <- "model {
  for(i in 1:N) {
    rsum[i] ~ dnorm(rtrue[i], prec.rsum[i])
    rtrue[i] ~ dnorm(mu[i], tau[1])
    mu[i] <- b.ss[ss[i]]
    loglik[i] <- logdensity.norm(rsum[i], rtrue[i], prec.rsum[i])
  }

  for(i in 1:N_ss) {
    b.ss[i] ~ dnorm(mu.ss[i], tau[2])
    mu.ss[i] <- b1 * tt[i]
  }

  b1 ~ dnorm(0, 0.1)

  for(i in 1:2) {
    tau[i] <- 1 / sigma2[i]
    sigma2[i] ~ dunif(0, 10)
  }

 }"
```

```
  write(mod, "model.txt")
  mod2 <- jags(model = "model.txt",
           data = list(rsum = rsum, tt = tt, N = N, prec.rsum = prec.rsum,
                    ss = ss, N_ss = N_ss),
           param = c("b1", "sigma2", "loglik"),
           n.chains = 3,
           n.iter = niter,
           n.burnin = nburn,
           parallel = T)

  mod2.sum <- mod2$summary
  mod2.sum[1:10, c(1,2,3,5,7,8,9)]

#################################################################################
# Model 3

  mod <- "model {
   for(i in 1:N) {
    rsum[i] ~ dnorm(rtrue[i], prec.rsum[i])
    rtrue[i] ~ dnorm(mu[i], tau[1])
    mu[i] <- b.ss[ss[i]]
    loglik[i] <- logdensity.norm(rsum[i], rtrue[i], prec.rsum[i])
   }

   for(i in 1:N_ss) {
    b.ss[i] ~ dnorm(mu.ss[i], tau.v[i])
    mu.ss[i] <- 0
    tau.v[i] <- 1 / v[i]
    v[i] <- sigma2.v + k * tt[i]
   }

   k ~ dnorm(0, 0.1)
   sigma2.v ~ dunif(0, 10)

   for(i in 1:1) {
    tau[i] <- 1 / sigma2[i]
    sigma2[i] ~ dunif(0, 10)
   }

  }"


  write(mod, "model.txt")
  mod3 <- jags(model = "model.txt",
           data = list(rsum = rsum, tt = tt, N = N, prec.rsum = prec.rsum,
                    ss = ss, N_ss = N_ss),
           param = c("sigma2", "sigma2.v", "k", "loglik"),
           n.chains = 3,
           n.iter = niter,
           n.burnin = nburn,
```

```
          parallel = T)

  mod3.sum <- mod3$summary
  mod3.sum[1:10, c(1,2,3,5,7,8,9)]

###################################################################################
# Model 4

  mod <- "model {
    for(i in 1:N) {
      rsum[i] ~ dnorm(rtrue[i], prec.rsum[i])
      rtrue[i] ~ dnorm(mu[i], tau[1])
      mu[i] <- b.ss[ss[i]]
      loglik[i] <- logdensity.norm(rsum[i], rtrue[i], prec.rsum[i])
    }

    for(i in 1:N_ss) {
      b.ss[i] ~ dnorm(mu.ss[i], tau.v[i])
      mu.ss[i] <- b1 * tt[i]
      tau.v[i] <- 1 / v[i]
      v[i] <- sigma2.v + k * tt[i]
    }

    b1 ~ dnorm(0, 0.1)
    k ~ dnorm(0, 0.1)
    sigma2.v ~ dunif(0, 10)

    for(i in 1:1) {
      tau[i] <- 1 / sigma2[i]
      sigma2[i] ~ dunif(0, 10)
    }

  }"


  write(mod, "model.txt")
  mod4 <- jags(model = "model.txt",
          data = list(rsum = rsum, tt = tt, N = N, prec.rsum = prec.rsum,
                  ss = ss, N_ss = N_ss),
          param = c("b1", "sigma2", "sigma2.v", "k", "loglik"),
          n.chains = 3,
          n.iter = niter,
          n.burnin = nburn,
          parallel = T)

  mod4.sum <- mod4$summary
  mod4.sum[1:10, c(1,2,3,5,7,8,9)]

###################################################################################
# Model 5
```

```
  mod <- "model {
   for(i in 1:N) {
    rsum[i] ~ dnorm(rtrue[i], prec.rsum[i])
    rtrue[i] ~ dnorm(mu[i], tau[1])
    mu[i] <- b.ss[ss[i]]
    loglik[i] <- logdensity.norm(rsum[i], rtrue[i], prec.rsum[i])
   }

   for(i in 1:N_ss) {
    b.ss[i] ~ dt(mu.ss[i], tau[2], nu[i])
    mu.ss[i] <- 0
    nu[i] <- n + k * tt[i]
   }

   k ~ dnorm(0, 0.1)
   n ~ dexp(1/30)

   for(i in 1:2) {
    tau[i] <- 1 / sigma2[i]
    sigma2[i] ~ dunif(0, 10)
   }

  }"


  write(mod, "model.txt")
  mod5 <- jags(model = "model.txt",
          data = list(rsum = rsum, tt = tt, N = N, prec.rsum = prec.rsum,
                  ss = ss, N_ss = N_ss),
          param = c("sigma2", "k", "n", "loglik"),
          n.chains = 3,
          n.iter = niter,
          n.burnin = nburn,
          parallel = T)

  mod5.sum <- mod5$summary
  mod5.sum[1:10, c(1,2,3,5,7,8,9)]

#########################################################################################
# Model 6

  mod <- "model {
   for(i in 1:N) {
    rsum[i] ~ dnorm(rtrue[i], prec.rsum[i])
    rtrue[i] ~ dnorm(mu[i], tau[1])
    mu[i] <- b.ss[ss[i]]
    loglik[i] <- logdensity.norm(rsum[i], rtrue[i], prec.rsum[i])
   }

   for(i in 1:N_ss) {
    b.ss[i] ~ dt(mu.ss[i], tau[2], nu[i])
```

```
    mu.ss[i] <- b1 * tt[i]
    nu[i] <- n + k * tt[i]
  }

  b1 ~ dnorm(0, 0.1)
  k ~ dnorm(0, 0.1)
  n ~ dexp(1/30)

  for(i in 1:2) {
    tau[i] <- 1 / sigma2[i]
    sigma2[i] ~ dunif(0, 10)
  }

}"


write(mod, "model.txt")
mod6 <- jags(model = "model.txt",
      data = list(rsum = rsum, tt = tt, N = N, prec.rsum = prec.rsum,
            ss = ss, N_ss = N_ss),
      param = c("b1", "sigma2", "k", "n", "loglik"),
      n.chains = 3,
      n.iter = niter,
      n.burnin = nburn,
      parallel = T)

mod6.sum <- mod6$summary
mod6.sum[1:10, c(1,2,3,5,7,8,9)]

#####################################################################################
# Model variation in mean among family pairs

# family pair for each study x species pair
 a <- table(ss, dat$ff)
 ff <- apply(a, 1, function(x) which(x > 0))
 N_ff <- max(ff)

# Model 7
 mod <- "model {
  for(i in 1:N) {
    rsum[i] ~ dnorm(rtrue[i], prec.rsum[i])
    rtrue[i] ~ dnorm(mu[i], tau[1])
    mu[i] <- b.ss[ss[i]]
    loglik[i] <- logdensity.norm(rsum[i], rtrue[i], prec.rsum[i])
  }

  for(i in 1:N_ss) {
    b.ss[i] ~ dt(mu.ss[i], tau[2], nu[i])
    mu.ss[i] <- b1 * tt[i] + b.ff[ff[i]]
    nu[i] <- n[1] + k * tt[i]
  }
```

```
  for(i in 1:N_ff) {
    b.ff[i] ~ dt(0, tau[3], n[2])
  }

  n[1] ~ dexp(1/30)
  n[2] ~ dexp(1/30)
  b1 ~ dnorm(0, 0.1)
  k ~ dnorm(0, 0.1)

  for(i in 1:3) {
    tau[i] <- 1 / sigma2[i]
    sigma2[i] ~ dunif(0, 10)
  }

}"


 write(mod, "model.txt")
 mod7 <- jags(model = "model.txt",
         data = list(rsum = rsum, tt = tt, N = N, prec.rsum = prec.rsum,
                 ff = ff, N_ff = N_ff,
                 ss = ss, N_ss = N_ss),
         param = c("b1", "k", "n", "sigma2", "b.ff", "loglik"),
         n.chains = 3,
         n.iter = 15000,
         n.burnin = 5000,
         parallel = T,
         seed = 456)

 mod7.sum <- mod7$summary
 mod7.sum[1:10, c(1,2,3,5,7,8,9)]

#####################################################################################
######
# compute LOO values
 llist <- list(mod1$sims.list$loglik, mod2$sims.list$loglik, mod3$sims.list$loglik,
mod4$sims.list$loglik,
          mod5$sims.list$loglik, mod6$sims.list$loglik, mod7$sims.list$loglik)

 w <- list()
 for(i in 1:7) {
  w[[i]] <- loo(llist[[i]])
 }

# compare models
 cm <- compare(w[[1]], w[[2]], w[[3]], w[[4]], w[[5]], w[[6]], w[[7]])
 cm

 out.loo <- data.frame(model = substr(rownames(cm), 4, 4),
             loo = round(cm[, 2] * -2, 1),
```

```
                    loo_dif = round(cm[, 1] * -2, 1),
                    loo_se = round(cm[, 3] * 2, 1))

  out.loo <- arrange(out.loo, loo_dif)
  out.loo

##########################################################################################
# density function compatible with the t-distribution in JAGS
  dens_t <- function(x, mu, s2, nu) {
        tau <- 1 / s2
        a <- gamma((nu+1)/2) / gamma(nu/2)
        b <- (tau / (nu * pi))^0.5
        c <- (1 + (tau * (x - mu)^2) / nu)^(-(nu + 1) / 2)
        d <- a*b*c
        return(d)
       }

# function to generate random values from t-distribution
# t-distribution is a mixture of normals with common mean and precision that is gamma distributed
  ran_t <- function(n, mu, s2, nu) {
    # generate values of precision drawn from a gamma distribution
    tau <- rgamma(n, shape = nu/2, rate = (s2*nu)/2)
    # convert precision to standard deviation
    sigma <- sqrt(1 / tau)
    # generate draws from a normal distribution with mean 0 and precision drawn from a gamma
distribution
    y <- rnorm(n, mean = mu, sd = sigma)
    return(y)
  }

##########################################################################################
# check the functions work
  hist(ran_t(10000, mu = 0, s2 = 0.01, nu = 7), breaks = 40, freq = F)
  curve(dens_t(x, mu = 0, s2 = 0.01, nu = 7), -20, 20, add = T, n = 1000)

##########################################################################################
# Figure 4
# split for within and among families
  a <- 1
  a <- ifelse(dat$Phylogenetic.distance > 360, 2, a)
  table(a)

  m <- tapply(dat$Phylogenetic.distance, a, median)
  ua <- unique(a)
  maint <- c("0-360 myr", ">360 myr")
  panel_lab <- c("A", "B")

  pdf("Figure 4.pdf")

  par(mfrow = c(2, 2), mar = c(4, 4, 2, 1))
```

```
for(i in 1:length(ua)) {

if(i %in% c(1)) yl <- "Density" else yl <- ""
if(i %in% c(1, 2)) xl <- expression(italic(r)) else xl <- ""

hist(dat$rsum[a == ua[i]],
    breaks = seq(-5, 15, 0.2), freq = F, ylim = c(0, 1), xlim = c(-3, 3),
    col = "grey", border = "grey", main = maint[i],
    xlab = xl, ylab = yl,
    yaxt = "n", cex.lab = 1.2)
 mtext(expr(bold(!!panel_lab[i])), side=3, adj=-0.05, line=0.5, cex = 1.2)

if(i %in% c(1, 3)) axis(2, at = c(0, 0.4, 0.8, 1.2), labels = c("0", "0.4", "0.8", "1.2")) else {
            axis(2, at = c(0, 0.4, 0.8, 1.2), labels = c("", "", "", "")) }

b1 <- mod4.sum[1, 1]
# sum variance terms
sigma2 <- sqrt(mod4.sum[2, 1]^2 + mod4.sum[3, 1]^2)
k <- mod4.sum[4, 1]

v <- sigma2 + k * m[i]/100
s <- sqrt(v)
b <- b1 * m[i]/100

curve(dnorm(x, b, s), -5, 15, add = T, lwd = 2, n = 1000)

b1 <- mod6.sum[1, 1]
sigma2 <- sqrt(mod6.sum[2, 1]^2 + mod6.sum[3, 1]^2)
k <- mod6.sum[4, 1]
n <- mod6.sum[5, 1]

d <- n + k * m[i]/100
b <- b1 * m[i]/100

curve(dens_t(x, b, sigma2, d), -5, 15, add = T, lwd = 2, n = 1000, col = "red")
abline(v = b, lwd = 2, lty = 2, col = "red")

# variance of the data
dd <- round(var(dat$rsum[a == ua[i]]), 2)
text(2, 0.8, paste("Variance = ", dd), xpd = NA, cex = 1.2)
}

dev.off()

##########################################################################################
# Figure 3
# plot parameter estimates

pe <- data.frame(model = c("Model 2", "Model 3", "Model 4", "Model 5", "Model 6", "Model 7"),
          b = c(mod2.sum[1, 1], 0, mod4.sum[1, 1], 0, mod6.sum[1, 1], mod7.sum[1, 1]),
          b_lcl = c(mod2.sum[1, 3], 0, mod4.sum[1, 3], 0, mod6.sum[1, 3], mod7.sum[1, 3]),
```

```
              b_ucl = c(mod2.sum[1, 7], 0, mod4.sum[1, 7], 0, mod6.sum[1, 7], mod7.sum[1, 7]),
              k = c(0, mod3.sum[3, 1], mod4.sum[4, 1], mod5.sum[3, 1], mod6.sum[4, 1], mod7.sum[2,
1]),
              k_lcl = c(0, mod3.sum[3, 3], mod4.sum[4, 3], mod5.sum[3, 3], mod6.sum[4, 3],
mod7.sum[2, 3]),
              k_ucl = c(0, mod3.sum[3, 7], mod4.sum[4, 7], mod5.sum[3, 7], mod6.sum[4, 7],
mod7.sum[2, 7]))

  pdf("Figure 3.pdf")

  par(mfrow = c(1, 2), mar = c(8, 5, 2, 1))
  xx <- 1:6
  yl <- c(min(pe$b_lcl, na.rm = T), max(pe$b_ucl, na.rm = T))
  plot(b ~ xx, data = pe, ylim = yl, xlim = c(0, 6), xaxt = "n", xlab = "", type = "n", bty = "l",
      ylab = expression(italic(beta)), cex.lab = 1.3)
    arrows(xx, pe$b_lcl, xx, pe$b_ucl, length = 0, lwd = 2, col = "grey")
    points(b ~ xx, data = pe, pch = 19, cex = 2)
    abline(h = 0, lwd = 2, lty = 2)
    axis(1, at = 1:6, labels = rep("", 6))
    text(xx + 0.5, -0.042, pe$model, srt = 50, xpd = NA, cex = 1.2, pos = 2)
    mtext(expr(bold(A)), side=3, adj=-0.05, line=0.5, cex = 1.2)

  yl <- c(min(pe$k_lcl, na.rm = T), max(pe$k_ucl, na.rm = T))
  plot(k ~ xx, data = pe, ylim = yl, xlim = c(0, 6), xaxt = "n", xlab = "", type = "n", bty = "l",
      ylab = expression(italic(k)), cex.lab = 1.3)
    arrows(xx, pe$k_lcl, xx, pe$k_ucl, length = 0, lwd = 2, col = "grey")
    points(k ~ xx, data = pe, pch = 19, cex = 2)
    abline(h = 0, lwd = 2, lty = 2)
    axis(1, at = 1:6, labels = rep("", 6))
    text(xx + 0.5, -1, pe$model, srt = 50, xpd = NA, cex = 1.2, pos = 2)
    mtext(expr(bold(B)), side=3, adj=-0.05, line=0.5, cex = 1.2)

  dev.off()

####################################################################################
# Figure 5
# Visualize results of Model 7
  b <- mod7.sum[substr(rownames(mod7.sum), 1, 4) == "b.ff", c(1, 3, 7)]
  b <- data.frame(b)
  names(b) <- c("b", "b_lcl", "b_ucl")
  b$fam <- levels(factor(dat$ff))
  b$same_fam <- ifelse(table(dat$ff, dat$same_fam)[, 2] > 0, 1, 0)
  b$n_pair <- table(dat$ff)
  b$pd <- tapply(dat$Phylogenetic.distance, dat$ff, mean)

# sig different from mean
  b$sigu <- ifelse(b$b_lcl > 0, 1, 0)
  b$sigl <- ifelse(b$b_ucl < 0, 1, 0)
  b$sig <- b$sigu + b$sigl

  b <- arrange(b, -b)
```

```
  b

  cl <- rgb(0, 0, 0, 0.5)
  cl <- ifelse(b$sig == 1, rgb(1, 0, 0, 0.5), cl)

  pdf("Figure 5.pdf")

  par(mfrow = c(1, 1), mar = c(5, 5, 1, 1))
  plot(b$b ~ jitter(b$pd, 200), pch = 19, cex = 1.5, col = cl, bty = "l",
      xlab = "Phylogenetic distance (myr)", ylab = "Family pair deviations from overall mean", cex.lab =
1.3)
    abline(h = 0)
    abline(v = 350, lty = 2)

# plot names
# use abbrev
  a1 <- substr(unlist(lapply(strsplit(b$fam, split = " "), function(x) x[1])), 1, 3)
  a2 <- substr(unlist(lapply(strsplit(b$fam, split = " "), function(x) x[2])), 1, 3)
  b$fam_abb <- paste(a1, "-", a2, sep = "")

  ab <- b %>%
      filter(sig == 1)

  p <- rep(3, nrow(ab))
  p <- ifelse(ab$fam_abb == "Ast-Fab", 1, p)

  text(ab$pd, ab$b, labels = ab$fam_abb, pos = p)

  dev.off()

  ab[order(ab$fam_abb), ]

# families involved
  f1 <- unlist(lapply(strsplit(ab$fam, split = " "), function(x) x[1]))
  f2 <- unlist(lapply(strsplit(ab$fam, split = " "), function(x) x[2]))
  fam_list <- c(f1, f2)
  table(fam_list)
```
